# Evaluation of the HACCP System in a University Canteen: Microbiological Monitoring and Internal Auditing as Verification Tools

**DOI:** 10.3390/ijerph10041572

**Published:** 2013-04-17

**Authors:** Andrea Osimani, Lucia Aquilanti, Stefano Tavoletti, Francesca Clementi

**Affiliations:** Department of Agricultural, Food and Environmental Sciences, Polytechnic University of Marche, via Brecce Bianche, Ancona 60131, Italy; E-Mails: a.osimani@univpm.it (A.O.); s.tavoletti@univpm.it (S.T.); f.clementi@univpm.it (F.C.)

**Keywords:** food safety, microbial contamination, check-list, catering, staff training

## Abstract

Food safety is essential in mass catering. In Europe, Regulation (EC) No. 852/2004 requires food business operators to put in place, implement and maintain permanent procedures based on Hazard Analysis and Critical Control Point (HACCP) principles. Each HACCP plan is specifically implemented for the processing plant and processing methods and requires a systematic collection of data on the incidence, elimination, prevention, and reduction of risks. In this five-year-study, the effectiveness of the HACCP plan of a University canteen was verified through periodic internal auditing and microbiological monitoring of meals, small equipment, cooking tools, working surfaces, as well as hands and white coats of the canteen staff. The data obtained revealed no safety risks for the consumers, since *Escherichia coli*, *Salmonella* spp. and *Listeria monocytogenes* were never detected; however, a quite discontinuous microbiological quality of meals was revealed. The fluctuations in the microbial loads of mesophilic aerobes, coliforms, *Staphylococcus aureus*, *Bacillus cereus*, and sulphite-reducing clostridia were mainly ascribed to inadequate handling or processing procedures, thus suggesting the need for an enhancement of staff training activities and for a reorganization of tasks. Due to the wide variety of the fields covered by internal auditing, the full conformance to all the requirements was never achieved, though high scores, determined by assigning one point to each answer which matched with the requirements, were achieved in all the years.

## 1. Introduction

Food safety is an increasingly important public health issue, and it is essential in mass catering establishments due to the enormous amount of meals served each day worldwide in childcare, schools, hospitals, businesses and nursing home canteens [1]. Nevertheless, foodborne outbreaks caused by mass caterers are still being reported [2]. Foodborne diseases encompass a wide spectrum of illnesses as a result of the ingestion of foodstuffs contaminated with microorganisms or chemicals. The occurrence of pathogens in foods may be caused by heavy microbial contamination, due to cross-contamination (insufficient hygiene of the staff and the environment), and/or by improper conditions enabling growth or survival of microorganisms, like temperature abuse or inadequate cooking, the latter especially for poultry, pork, burgers, sausages and kebabs. A combination of the factors listed above, leading to general unsanitary conditions [3,4].

As a repercussion of the Bovine Spongiform Encephalopathy (BSE) crisis and several other food scandals, the European Union decided to promote an action plan for a pro-active new food policy, with traceability as a basic concept. In January 2002, the EU adopted the framework legislation in Regulation (EC) No 178/2002 laying down the general principles and requirements of EU food law [5]; in April 2004, the EU adopted three basic acts forming the core of the so-called “Food Hygiene Package” provided for in the following key acts: Regulation (EC) 852/2004 on the hygiene of foodstuffs [6] which replaced Directive 93/43/EEC; Regulation (EC) 853/2004 laying down specific hygiene rules for food of animal origin [7], and Regulation (EC) 854/2004 laying down specific rules for the organization of official controls on products of animal origin intended for human consumption [8]. In addition, from 1 January 2006, EC Regulation 2073/2005, and subsequent amendments, established the microbiological criteria for some food-borne bacteria, for microbial toxins and metabolites [9,10]. Article 5 of Regulation (EC) No 852/2004 requires food business operators to put in place, implement and maintain a permanent procedure based on Hazard Analysis and Critical Control Point (HACCP) principles. The HACCP system is the internationally agreed approach to food safety control; it must be applied throughout the food chain from primary production to final consumption and its implementation should be guided by scientific evidence of risks to human health. 

A valid HACCP program requires a methodical collection of consistent data on the incidence, elimination, prevention, and reduction of risks. 

Microbiological analyses are an important tool to collect data to be used for the development and verification of an HACCP plan [1,11,12,13,14] as well as to assess the effectiveness of sanitation operations, to evaluate the compliance of incoming ingredients with safety criteria, and to determine the safety of end products [15]. In addition to these operations, periodic audits can be undoubtedly useful to examine the application of all control measures foreseen by an HACCP plan [16,17]. As stated by the ISO standards, an audit is a systematic and independent examination to verify whether activities and results comply with the documented procedures and also whether these procedures are implemented effectively to achieve the objectives. Internal audits can contribute to the reduction in health risks as it is a powerful tool for catering companies to improve safe food production, even though some limitations are still present. 

In this study, the effectiveness of the HACCP plan of a University canteen that produces up to 1,200 meals a day was verified over five years (2008–2012) through the microbiological monitoring of meals, small equipment, cooking tools, tableware, chopping boards, as well as hands and white coats of the canteen staff. During the monitoring period, two internal audits each year were also carried out in order to determine the correct execution of the procedures foreseen by the HACCP plan. 

## 2. Experimental Section

The HACCP system was applied in accordance with the mandatory provisions of European Regulation (EC) 852/2004 [6]. The implementation process of the HACCP system followed the five preliminary steps, namely: (i) formation of the HACCP team; (ii) description of products; (iii) identification of the intended use; (iv) construction of flow charts; (v) on-site confirmation of flow charts; and the seven principles, namely: (i) identification of hazards; (ii) determination of critical control points (CCP); (iii) identification of critical limits; (iv) setting-up of monitoring procedures; (v) organisation of corrective actions; (vi) setting-up of procedures to verify that the HACCP system was working as intended; and (vii) organisation of record-keeping procedures. 

### 2.1. Description of the Canteen

The University canteen, already described by Osimani *et al.* 2011 [1], is organized in different rooms, namely offices, warehouse, utensil and pan storage room, chemical storage room, local water treatment network, dressing rooms and toilets for the staff, dish washing area, disposal of wastes, food and beverage distribution area, dining room, kitchen, frozen food cold room, fresh meat cold room, cheeses and fermented sausage cold room, fruit and vegetable cold room, receiving area for raw materials, and food preparation room; in order to avoid the risk of cross-contamination, the food preparation room is divided in different preparation areas, namely uncooked red meats, white meats, fish, vegetables, cheeses and fermented sausages. 

Meat-, fish- and vegetable-based meals are prepared during the morning and stored in hold-hot apparatus or cooled in blast chillers when necessary, while pasta-based meals are prepared quickly using an express “cook-served” service system. All the meals are stored in stainless steel boxes, at the proper temperature, which is between +60 and +65 °C for the warm-served meals and below +10 °C for the cold-served and gastronomic meals. The leftovers of the lunch are cooled in a blast chiller, stored in a refrigerator and administered at dinner on the same day of preparation. The canteen is certified with the ISO 9001:2000/2008 quality mark [18] since 2008.

### 2.2. Food Analyses

All the meals were sampled with a casual frequency and with no prior notice, using sterile instruments and bags (Sto-Circul-Bag, Pbi International, Milan, Italy). They were kept under refrigerated conditions and subjected to microbiological analyses within 2 h of collection. 

Three categories of products were sampled: (i) cooked and warm-served products (referred to as “w”), (ii) cooked and cold-served products (referred to as “c”) and (iii) cold gastronomic products, which could contain raw ingredients ready for consumption, (referred to as “g”).

For each year, 17 food samples, which were representative of the meals prepared in the canteen, were analyzed. Cooked and warm-served products (40 samples) included: pasta with tomato, meat sauce, or aubergines, green beans, beans with tomato, peas, lentils, boiled potatoes, chards and potatoes, roast pork and roast chicken, veal cooked with red wine, omelettes with spinach, flounder with lemon, meat roll and meat balls with potatoes, sole, cuttlefish with peas, roast dogfish, and roast hake.

Cooked and cold-served products (18 samples) included: seafood salad, green beans, boiled meat dressed with green sauce (parsley, capers, pickled gherkins, anchovy) or “*aurora* sauce” (thin broth, tomato purée, cream), boiled meat with raw apples, boiled meat with carrots, boiled Savoy cabbage, and sliced turkey. 

Finally, cold gastronomic products (27 samples) included: green, mixed, or “*caprese*” (hand-cut fresh tomatoes, mozzarella cheese, and basil) salad, ham, emmenthal cheese, and raw savoy cabbage.

Within each food category, some food preparations (e.g., boiled meat dressed with green sauce, green salad, *etc.*) were repeatedly sampled during the study.

A total of 179 food preparations, including those subjected to microbiological analyses, underwent temperature monitoring using a high precision thermometer “Checktemp 98509-1” (Hanna Instruments, Milan, Italy). The acceptability of meals using this parameter was established on the basis of D.P.R. no. 327 of 26/03/1980 (Art. 31), published in the Official Gazette of the Italian Republic No. 193 of 16/07/1980 [19].

Twenty-five grams of each sample were aseptically weighed in sterile bags, diluted in 225 mL of peptone water (bacteriological peptone 1 g/L) and homogenized in a Stomacher 400 Circulator apparatus (PBI International, Milan, Italy) for the enumeration of the following microorganisms: total mesophilic aerobes (TMA), coliforms (C), *Escherichia coli* (Ec), *Staphylococcus aureus* (Sa), *Bacillus cereus* (Bc), and sulphite-reducing clostridia (SRC).

All the samples were analysed as already described by Osimani *et al.* [1]. Briefly, total mesophilic aerobes were counted in Standard Plate Count Agar (PCA, Oxoid, Basingstoke, UK); *E. coli* and coliforms in Chromogenic Coliform Agar (CCA, Biolife, Milan, Italy); *B. cereus* on *Bacillus cereus* Agar Base added with antimicrobic supplement (Biolife, Milan, Italy); sulphite-reducing clostridia in Tryptose Sulphite Cycloserine (TSC, Biolife); *S. aureus* on Baird Parker Agar Base supplemented with Egg-Yolk Tellurite Emulsion (Oxoid). For the enumeration of the latter microorganism, black colonies with convex elevation and light halo underwent the Staphylase test (Oxoid) and those positive for coagulase reaction were further subjected to *t*-test (Oxoid). The presence of *Listeria monocytogenes* and *Salmonella* spp. was assessed in accordance with AFNOR BIO 12/11-03/04 [20] and AFNOR BIO 12/16-09/05 [21] standard methods, respectively.

Acceptability of food samples was arbitrarily established on the basis of the microbiological limits set by the Italian guidelines for food product microbiological quality as previously reported by Osimani *et al.* 2011 [1] and shown in Table 1.

### 2.3. Environmental Analyses

Small equipment, cooking tools, tableware, and chopping boards were microbiologically examined using the Swab Rince Kit (Oxoid); *E. coli*, coliforms and total mesophilic aerobes were further enumerated as previously described by Osimani *et al.* [1]. Small equipment and cooking tools (referred to as “e”) (32 swabs) included: slicing machines, meat grinder, cutlery and pans; tableware (referred to as “t”) (18 swabs) included: stainless steel preparation tables and shelves; chopping boards (referred to as “b”) (25 swabs) included: a raw meat chopping board, a fresh vegetable chopping board and a raw fish chopping board. All the surface sampling procedures were performed in the absence of the canteen staff in order to verify the appropriateness of cleaning and sanitation operations carried out at the end of the day of work. The hygiene of the canteen staff was also monitored by surface-swabbing of hands and white coats for the enumeration of *E. coli*, coliforms and *S. aureus*, as previously described by Osimani *et al.* 2011 [1]. Acceptability of surface and staff hygiene was arbitrarily established on the basis of the microbiological limits reported by Osimani *et al.* [1]; the microbial limits adopted for the acceptability of surfaces were: 1.0 Log·cfu/cm^2^ for total mesophilic aerobes, 0 Log·cfu/cm^2^ for coliforms and *E. coli*. Microbial limits adopted for the acceptability of hands and white coats swab samples were: 1.0 Log·cfu/cm^2^ for coliforms and, 1.0 Log·cfu/cm^2^ for *S. aureus*.

**Table 1:** Microbiological limits for food samples.

Sample	TMA	C	Ec	Sa	Bc	SRC	S	L
	Log cfu/g	Log cfu/g	Log cfu/g	Log cfu/g	Log cfu/g	Log cfu/g		
Cooked products	4.0	3.0	1.0	2.0	2.0	1.0	Abs./25g	Abs./25 g
(warm- and cold- served)								
Cold gastronomic products	5.7	3.0	1.0	2.0	2.0	1.0	Abs./25 g	Abs./25 g

Bacterial class codes: TMA = Total Mesophilic Aerobes; C = Coliforms; Ec = *Escherichia coli*; Sa = *Staphylococcus aureus*; Bc = *Bacillus cereus;* SRC = Sulphite-Reducing Clostridia; S = *Salmonella* spp.; L = *Listeria monocytogenes*; cfu: colony forming units; Abs: absence.

### 2.4. Audit

Two internal audits per year were performed from 2008 to 2012 by a trained advisor to determine whether selected activities of the HACCP systems and the related results complied with planned arrangements and whether these arrangements were suitable and implemented effectively in order to achieve food safety objectives. Inspections were always performed in the morning with a casual frequency, with no prior notice and during the food preparation activities. Each inspection covered the main areas of the canteen, namely: (i) raw materials receiving area; (ii) preparation areas; (iii) cold rooms; (iv) warehouse; (v) kitchen; and (vi) food and beverage distribution area. 

The audit check-list is shown in Table 2; check-list items were chosen by the advisor in agreement with the HACCP coordinator and the HACCP team on the basis of the HACCP plan. For all the questions reported in the check-list, an answer in terms of “yes” or “no” was assigned. Non-conformance was adjudged to questions with negative answers. At the end of each audit, a report, summarizing all the non-conformances, was filled out. Annual scores were determined by assigning one point to each answer which matched with the requirements; a maximum of 72 points per year (resulting from 36 questions per audit; two audits per year) was achievable.

**Table 2:** Audit check-list and results for the attribution of non-conformances from 2008 to 2012.

Plant name:		Years									
Date: hours:		2008		2009		2010		2011		2012	
**Items**		Y	N	Y	N	Y	N	Y	N	Y	N
**1. Raw material receiving area**											
1a	Is the receiving area clean and clear from packing boxes?	●●		●●		●●		●●		●●	
1b	Are perishable foods quickly stored in refrigerated conditions as soon as they arrive?	●●		●●		●●		●●		●●	
**2. Food storage**											
2a*	Do the temperatures of refrigerators, freezers and cold rooms comply with the standards?	●●		●●		●●		●●		●●	
**3. General hygiene**											
3a	Are cooked and raw products always separated?	●●		●●		●●		●●		●●	
3b	Are spoiled products present?	●●		●●		●●		●●		●	●
3c	Are non-conforming products clearly identified?	●●		●●		●●		●	●	●●	
3d	Are expired foods absent?	●●		●	●	●●		●●		●●	
3e	Are all food products stored without letting them touch the floor?	●●		●●		●	●	●	●	●	●
3f	Are the shelves clean?	●●		●●		●●		●●		●●	
3g	Are all rooms visibly clean?	●●		●●		●●		●●		●●	
3h	Are pests absent?	●●		●●		●●		●●		●	●
**4. Food preparation areas**											
4a	Are all foods left at room temperature as required?	●●		●●		●●		●●		●●	
4b	Is cross-contamination avoided?	●●		●●		●●		●●		●●	
4c	Are cooked and cold-served products cooled at 10 °C within a maximum of 4 h?	●●		●●		●●		●●		●●	
4d*	Are foods defrosted in the proper way?	●●		●●		●●		●●		●●	
4e	Are chopping boards and knives adequately clean?	●●		●●		●●		●●		●●	
4f	Is the extractor fan clean?	●●		●●		●●		●●		●●	
4g	Is the tableware clean?	●●		●●		●●		●●		●●	
**5. Food distribution area**											
5a	Is cooked and warm-served food temperature between 60 and 65 °C?	●●		●●		●●		●●		●●	
5b*	Is cooked and cold-served food temperature less than 10 °C?	●	●		●●	●	●		●●		●●
5c	Are self-service desks clean?	●●		●●		●●		●●		●●	
**6. Staff hygiene**											
6a	Is the ban on wearing earrings, necklaces, and watches, and on smoking and eating obeyed?		●●	●	●	●	●	●●		●●	
6b	Are the hands clean?	●●		●●		●●		●●		●●	
6c	Are the wounds adequately protected?	●●		●●		●●		●●		●●	
6d	Are the white coats clean?	●●		●●		●●		●●		●●	
6e	Is the cap correctly worn?	●●		●●		●●		●●		●●	
6f	Are service shoes clean?	●●		●●		●●		●●		●●	
6g	Are smoking and eating bans obeyed?	●●		●●		●●		●●		●●	
6h	Are protective gloves adequately worn?	●●		●●		●●		●●		●●	
**7. Registrations**											
7a	Has the non-conformance/corrective action log been filled out?	●●		●●			●●	●●		●●	
7b	Has the cooked and warm-served food temperature log been filled out?	●●		●●		●●		●●		●●	
7c	Has the cold-served food temperature log been filled out?	●●		●●		●●		●●		●●	
7d	Has the raw material log been filled out?	●●		●●		●●		●●		●●	
7e	Has the cold room temperature log been filled out?	●●		●●		●●		●●		●●	
7f	Has the deratization log been filled out?	●●		●	●	●	●	●●		●●	
7g	Has the traceability log been filled out?	●●		●●		●●		●●		●●	
**Annual scores **		**69/72**		**67/72**		**66/72**		**68/72**		**67/72**	

For each year, two audits were carried out; the symbol (●) represents the answer to each item for each audit. The annual score is the ratio between the number of answers matching the requirements (Y) and the total number of answers recorded (36 questions × 2 audits). Y: Yes, N: No. * Critical Control Point (CCP).

### 2.5. Statistical Analysis

Microbiological data concerning 27 variables obtained by combining categories (w, c, g, e, t, b) with bacterial classes (TMA, C, Ec, Sa, Bc, SRC) were collected. Each variable was expressed as percentage of conforming or non-conforming samples detected during the 2008–2012 period of time. A data matrix of the percentage of non-conforming samples obtained for each year-variable combination (5 years × 27 variables data matrix) was made. However, variables showing a complete absence of non-conforming samples across the 2008–2012 period were excluded from the statistical analysis carried out by Principal Component Analysis (PCA) using the Pearson Correlation matrix and the NTSYS software (Applied Biostatistics Inc., Port Jefferson, NY, USA).

## 3. Results and Discussion

Percentages of non-conforming samples defined on the basis of the temperature limits set for food kept under either hot or refrigerated conditions are shown in Table 3. The temperature recorded for non-conforming cooked and warm-served products (w) ranged between +49 °C and +59 °C; the highest number of non-conforming samples was found among cooked and cold-served products (c) whose temperature ranged between +13 °C and +24 °C and cold gastronomic products (g) whose temperature ranged between +12 °C and +22 °C. These findings suggest that more attention must be paid to the timing of food preparation and to the calibration of the equipment used for foods storage under refrigerated conditions. 

**Table 3:** Percentages of non-conforming samples based on their temperature maintenance.

Year	Total samples	Cooked and warm-served products (w)	Cooked and cold-served products (c)	Cold gastronomic products (g)	Total non-conforming samples
2008	39	1 out of 23 (4.3%)	1 out of 1 (100.0%)	4 out of 15 (26.7%)	6 (15.4%)
2009	40	1 out of 22 (4.5%)	4 out of 8 (50.0%)	6 out of 10 (60.0%)	11 (27.5%)
2010	34	3 out of 21 (14.3%)	3 out of 3 (100.0%)	7 out of 10 (70.0%)	13 (38.2%)
2011	33	1 out of 16 (6.2%)	4 out of 4 (100.0%)	9 out of 13 (69.2%)	14 (42.4%)
2012	33	0 out of 16	3 out of 6 (50.0%)	7 out of 11 (63.6%)	10 (30.3%)

Temperature acceptance limits: +60 °C ≤ T *<* +65 °C for cooked and warm-served products; T ≤ +10 °C for cooked and cold-served products and cold gastronomic products. The acceptability of food samples was established on the basis of D.P.R. No. 327 of the 26/03/1980 (Art. 31), published in the Official Gazette of the Italian Republic No. 193 of the 16/07/1980. In round brackets, percentages of non-conforming samples within each food category are reported, whereas in squared brackets, percentages of total non-conforming samples are reported.

Microbiological analysis of food samples revealed that there were no safety risks associated to the occurrence of *E. coli*, *Salmonella* spp. or *Listeria monocytogenes* in the meals served at the University canteen*,* but fluctuations in the load of total mesophilic aerobes, coliforms, *B. cereus*, *S. aureus*, and sulphite-reducing clostridia were seen. By contrast, superficial swabbing of hands and white coats revealed a high level of hygiene of the canteen staff. 

The results of the statistical analyses are summarized as follows. PCA identified three principal components that explained 88.84% of the total variance, 71.82% being explained by the first two principal components. These results reflected a high correlation among the 13 variables and showed a clear description of the change of microbiological contamination from 2008 to 2012 in the canteen that was analyzed.

The first principal component (PC1) explained 42.58% of the overall variance and was characterized by high and positive eigenvector coefficients for six out of the 13 variables included in the analysis (Table 4). The second principal component (PC2) explained 29.24% of the total variance and five variables were characterized by high positive (TMA-c, Bc-w, Bc-c, and Bc-g) or negative (TMA-g) eigenvector coefficients. Eigenvector, corresponding to the third principal component (PC3), is reported in Table 5, even though it was not considered for the subsequent evaluation of PCA results since most of the total variance was clearly explained by PC1 and PC2. However, the most important variables of PC3 were TMA-w and Sa-g with high positive coefficients (Table 5). The microbiological variable TMA-t did not show effective eigenvector coefficients for any principal component.

**Table 4:** Percentage of explained variance and eigenvector coefficients concerning the three main principal components.

Bacterial class	PC1 (42.58% *)	PC2 (29.24% *)	PC3 (17.02% *)
TMA-w	0.1499	0.0676	0.9860
TMA-c	0.0070	0.8102	0.0282
TMA-g	0.1147	−0.9852	0.1234
C-c	0.9757	−0.1668	−0.1074
C-g	0.9326	0.3430	−0.0915
Sa-g	0.3589	−0.3090	0.8286
Bc-w	−0.1925	0.9232	0.2168
Bc-c	−0.5870	0.6844	−0.1316
Bc-g	0.6942	0.6773	0.2127
SRC-w	0.7892	−0.0137	−0.6083
TMA-e	0.9797	0.1672	−0.0387
TMA-b	0.9200	0.1556	0.1420
TMA-t	0.5076	−0.3104	−0.1287

Bacterial class codes: TMA = Total Mesophilic Aerobes; C = Coliforms; Ec = *Escherichia coli*; Sa = *Staphylococcus aureus*; Bc = *Bacillus cereus;* SRC = Sulphite-Reducing Clostridia. Category codes: w = cooked and warm products; c = cooked and cold products; g = cold gastronomic products; e = small equipment and cooking tools; b = chopping boards; t = tableware. * Percentage of explained variance.

**Figure 1:**
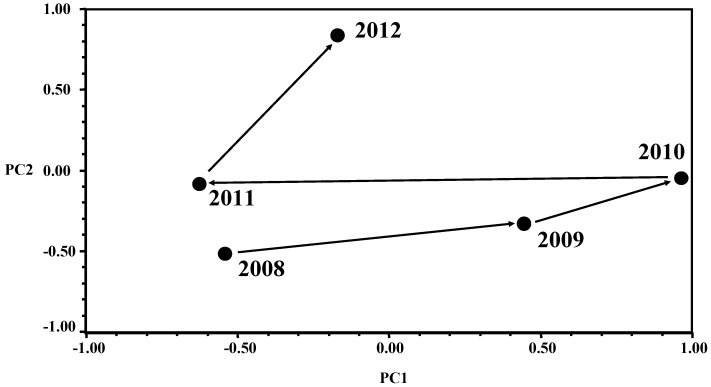
Results of Principal Component Analysis (PCA) showing the trend in the bacterial contamination of meals and surfaces sampled from 2008 to 2012 at the University canteen.

Overall PCA results can be interpreted based on the graph reported in Figure 1. Concerning PC1 scores, high score values (PC1 axis) mean high frequency of non-conforming samples for the combination of the 6 variables characterizing PC1 eigenvector (Table 4). Therefore, Figure 1 shows that the frequency of non-conforming samples for PC1 variables increased progressively from 2008 to 2010, decreased strongly in 2011 going back to the level of those in 2008, but again went up in 2012 even though at a lower effective level than in 2009 and 2010. These results indicate that the quality level of the canteen seems to have been unstable across the 2008–2012 period of time tested. The highest quality was obtained in 2008 and 2011 whereas 2009 and 2010 showed the worst non-conforming sample frequencies. The year 2012 however showed an intermediate behavior that was however closer to the 2008 and 2011 contamination levels than to the situation found in 2009 and 2010. 

**Table 5:** Frequency of non-conforming samples detected in 2008–2012 for each of the 13 microbiological variables used for the PCA. The variables are grouped based on their relative importance within each principal component. The sign of the eigenvector coefficient for each variable is also included.

Years	Variables												
	C-c ^†^	C-g ^†^	Bc-g ^†^	SRC-w ^†^	TMA-e ^†^	TMA-b ^†^	TMA-c ^†^	Bc-w ^†^	Bc-c ^†^	TMA-g ^†^	TMA-w ^†^	Sa-g ^†^	TMA-t ^†^
	PC1 * (+)	PC1 * (+)	PC1 * (+)	PC1 * (+)	PC1 *(+)	PC1 * (+)	PC2 * (+)	PC2 * (+)	PC2 * (+)	PC2 * (−)	PC3 * (+)	PC3 * (+)	-
2008	0.0	0.0	0.0	0.0	20.0	25.0	0.0	0.0	0.0	50.0	15.4	0.0	33.3
2009	20.0	25.0	25.0	0.0	50.0	50.0	33.3	0.0	0.0	50.0	60.0	25.0	50.0
2010	33.3	50.0	33.3	57.1	66.7	66.7	33.3	0.0	0.0	33.3	0.0	0.0	50.0
2011	0.0	0.0	0.0	0.0	25.0	0.0	50.0	0.0	25.0	33.3	0.0	0.0	50.0
2012	0.0	25.0	37.5	0.0	37.5	40.0	60.0	33.3	25.0	0.0	33.3	0.0	33.3

Bacterial class codes: TMA = Total Mesophilic Aerobes; C = Coliforms; Ec = *Escherichia coli*; Sa = *Staphylococcus aureus*; Bc = *Bacillus cereus;* SRC = Sulphite-Reducing Clostridia; Category codes: w = cooked and warm products; c = cooked and cold products; g = cold gastronomic products; e = small equipment and cooking tools; b = chopping boards; t = tableware; * PCA component assigned to the variable and sign (+/−) of the eigenvector coefficient; **^†^** Values are expressed as percentage.

The analysis of PC2 scores (PC2 axis in Figure 1) indicated a progressive worsening of the canteen when the four variables with high and positive PC2 eigenvector coefficient (TMA-c, Bc-w, Bc-c, and Bc-g) are considered. PC2 scores increased from 2008 to 2012, the last year being the worst. 

This comprehensive trend detected across the 2008–2012 period of time can be verified following the change in the non-conforming sample frequencies over time by grouping the 13 variables based on their relative importance within each principal component and by the sign (+/−) of the corresponding eigenvector coefficient, as reported in Table 5. It is interesting to note that for variable TMA-g, which shows a high negative PC2 eigenvector coefficient (Table 4), a progressive improvement in quality was recorded from 2008 to 2012, where 2012 was characterized by the absence of non-conforming samples. Moreover, Table 5 also shows that variable TMA-w showed an improvement in quality level in 2009–2010, compared to the quality level in 2008 and 2009, but the frequency of non-conforming samples increased again in 2012, confirming the instability in the performance of the canteen for most of the parameters analyzed. Quality for the Sa-g variable was almost always very high, whereas TMA-t always showed high levels of contamination throughout the whole five-year period investigated (Table 5).

As expressed by Surak and Wilson [22], HACCP auditing is more than just a collection of records, it is a pivotal part of HACCP verification process. In fact, audit results can provide input to corrective actions and the management review process [22]. 

As can be seen from the results shown in Table 2, audit scores ranged from 66/72 to 69/72 points. Due to the wide variety of questions, as expected, a full conformance with all the requirements was never achieved. The most frequent non-conformances dealt with general hygiene matters; in particular, question 3e, dealing with food storage, obtained negative answers at least once a year from 2010 to 2012. 

Another frequent non-conformance was adjudged to question 5b, dealing with the maintenance temperature of cold-served food: in this case, the requirement was not matched at least once a year, and up to twice a year in 2009, 2011 and 2012. This finding highlighted the need for more incisive corrective actions and, probably, for reconsideration about the amount of cold-served meals produced and served by the facility. 

It is useful to point out that throughout the period of study, the number of food preparations produced in the canteen progressively increased from 1,000 to 1,200, and this phenomenon coincided with a gradual reduction of staff due to retirement which has not been replaced, yet. Therefore, the results should also be considered in terms of the human factor which, in this case, played an important role that cannot be entirely neglected.

It is likely that the increase in the number of meals prepared and served *per* day together with the decrease in the number of staff members led to an inappropriate handling of cold food, as can be seen from Table 3, where the cold-served and gastronomic meals encountered the highest percentages of non-conforming samples. 

As concerns the hygiene of the canteen staff, non-conformances adjudged to question 6a went down progressively until they disappeared completely during the last two years (2011–2012) of study, thus demonstrating the appropriateness of the staff training activities.

As regards the remaining non-conformances, the sporadic occurrence of negative results (e.g*.*, items 3b, 3c, 3d, 7a, and 7f) suggests that these problems might easily be solved with more attention and involvement of the canteen staff.

The implementation of the HACCP system started in 1997 [1]; over the years, several corrective actions were carried out most of which undertaken between 2000 and 2007 [1,13]. 

In the period considered (2008–2012), only a few corrective actions were undertaken. Since structural weaknesses have not been highlighted, the main corrective action was related to staff training in order to improve food safety culture [16]. In accordance with Deliberation No. 2173 ME/SAN of 10/12/2002 of the Marche Region (Italy) [23], which bestows the employer with the responsibility of staff training, the canteen staff was subjected to undergo training two times a year. Each training session focused on a specific topic, in relation to the importance of food safety (e.g*.*, cleaning activities, food handling, foodborne diseases), the role and benefits of HACCP, legal obligations, the principles of HACCP, the practical application of HACCP and the role of internal audits [16]. Each training course concluded with a questionnaire that consisted of 10 questions which had to be answered in 30 min. During the training courses the results of the microbiological analyses and audits were always showed and debating was encouraged.

## 4. Conclusions

Although no risks associated with the occurrence of food-borne pathogens in the meals served from 2008–2012 were found, the microbiological quality of meals and the application of hygienic procedures appeared to be quite discontinuous, which can also be seen from the results of periodic audits. It is very likely that all the negative results could be imputable to a non-optimal organization of the canteen staff. In view of a continuous improvement in procedures, staff training activities and staff involvement should be enhanced [24], together with a possible reorganization of tasks which should be arranged in accordance with the HACCP team. Based on the results obtained, microbiological monitoring and internal audits showed to be powerful verification tools for a practical evaluation of the HACCP plan, which contributed to the revelation of an adverse drift in the overall quality of the canteen.
